# Cardiorenal Anemia Syndrome and Survival among Heart Failure Patients in Tanzania: A Prospective Cohort Study

**DOI:** 10.1186/s12872-017-0497-2

**Published:** 2017-02-14

**Authors:** Pedro Pallangyo, Francis Fredrick, Smita Bhalia, Paulina Nicholaus, Peter Kisenge, Benjamin Mtinangi, Mohamed Janabi, Stephen Humphrey

**Affiliations:** 1 Department of Cardiovascular Medicine, Jakaya Kikwete Cardiac Institute, P.O Box 65141, Dar es Salaam, Tanzania; 2 Muhimbili University of Health and Allied Sciences, P.O Box 65001, Dar es Salaam, Tanzania

**Keywords:** CRAS, Heart failure, Renal insufficiency, Anemia, Mortality

## Abstract

**Background:**

Cardiorenal anemia syndrome (CRAS) is an evolving global epidemic associated with increased morbimortality and cost of care. The management of patients with CRAS remains a challenging undertaking worldwide and the lack of evidence-based clinical guidelines adds to the challenge. We aimed to explore the prevalence and survival rates of heart failure patients with CRAS in Tanzania.

**Methods:**

We screened 789 patients and consecutively recruited 463 who met the inclusion criteria. Each participant underwent an interview, physical examination, anthropometric measurements, anemia, renal functions and echocardiographic assessment. All participants were followed until death or for up-to 180 days, whichever came first. Bivariate comparison and subsequent Cox proportional-hazards regression model were used to compare the CRAS and non-CRAS groups with respect to the primary end point.

**Results:**

The mean age of participants was 46.4 ± 18.9 years, and 56.5% were women. Overall, 51.9% of participants had renal insufficiency, 72.8% were anemic and 44.4% had CRAS. During a mean follow-up of 103 ± 75 days, 57.8% of participants died. Patients with CRAS displayed a higher mortality rate (73.5%) compared to those free of CRAS (45.8%), (*p* < 0.001). During multivariate analysis in a cox regression model of 21 potential predictors of mortality; renal dysfunction (HR 1.9; 95% CI 1.0–3.5; *p* = 0.03), severe anemia (HR 1.8; 95% CI 1.0–3.1; *p* = 0.04), hyponatremia (HR 2.2; 95% CI 1.3–3.7; *p* = 0.004) and rehospitalization (HR 4.3; 95% CI 2.2–8.4; *p* < 0.001) proved to be the strongest factors.

**Conclusion:**

Cardiorenal anemia syndrome is considerably prevalent and is associated with an increase in mortality amongst patients with heart failure. In view of this, timely, aggressive and collaborative measures to improve renal functions and/or correct anemia are crucial in the management of CRAS patients. Furthermore, these findings call for guideline committees to revise and/or develop evidence-based recommendations for management of patients with CRAS.

**Electronic supplementary material:**

The online version of this article (doi:10.1186/s12872-017-0497-2) contains supplementary material, which is available to authorized users.

## Background

Comorbid heart failure, renal failure and anemia commonly referred to as cardiorenal anemia syndrome (CRAS) is an evolving epidemic encountered by healthcare professionals worldwide. The interactive links between the CRAS triad are complex and multi-factorial with high potential for increased morbidity, mortality, complexity and cost of care. Studies have reported CRAS prevalence ranging from 19 to 62% and mortality rates of up-to 51% amongst heart failure patients [1–8].

The management of patients with CRAS remains a challenging undertaking largely due to the complexity and heterogeneity of this syndrome. Furthermore, the present clinical guidelines lack evidence-based recommendations for managing patients with CRAS. Despite the evidence of increasing hospitalizations due to heart failure and renal dysfunction, CRAS has not been thoroughly explored in Tanzania. To investigate on this subject, we performed a prospective cohort study to determine the prevalence and survival rates of heart failure patients with CRAS.

## Methods

### Recruitment process and definition of terms

From March through October 2014, we screened for and consecutively enrolled patients with clinical diagnosis of heart failure who were admitted to Muhimbili National Hospital and the Jakaya Kikwete Cardiac Institute in Dar es Salaam, Tanzania. Demographic, clinical, laboratory, and echocardiographic data were gathered during the hospital admission of enrollment. We employed the following prospective selection criteria. Patients were included if they had symptoms of heart failure as per the Framingham criteria and later patients underwent a 2-dimensional echocardiography for diagnosis confirmation. We used an ejection fraction (EF) of below 45% to denote a systolic dysfunction. Renal dysfunction and anemia were diagnosed using the serum creatinine (sCr) and hemoglobin (Hb) measurements respectively. We estimated the renal functions with the Modification of Diet in Renal Disease (MDRD) equation, where estimated glomerular filtration rate (eGFR) (mL/min/1.73 m^2^) = 186.3 × sCr - 1.154 × age - 0.203 × 0.742 (if female). No correction for race was necessary as all participants were of African descent. Renal dysfunction was defined by an eGFR value of <60 mL/min/1.73 m^2^. WHO criteria for anemia i.e. Hb concentration of <13.0 g/dL for males and <12.0 g/dL for females was used to diagnose anemia. Participants were categorized as having CRAS if they had both renal dysfunction and anemia.

### Follow-up and study outcomes

Follow-up continued through April 2015, data was censored at the time of death or after the completion of the 180-day follow-up period, whichever occurred first. Follow-up was conducted through weekly phone calls and a participant was deemed lost to follow-up when all of the 3 phone numbers given during enrollment were not reachable in 3 different occasion 7-days apart, with at least 3 attempts on each occasion. The primary outcome measure was the all-cause mortality within 180 days after enrollment.

### Statistical analysis

STATA v11.0 software was utilized in all statistical analyses. We compared categorical variables using the Pearson Chi square tests or Fisher’s exact tests; Student’s T-test was used in comparison of continuous variables. Bivariate comparison and subsequent Cox proportional-hazards regression model were used to compare the CRAS and non-CRAS groups with respect to the primary end point. Statistically significant variables maintained in the regression final model underwent stepwise and backward selection procedures. The multivariate models were fitted with baseline covariates associated with mortality by bivariate analysis at the <0.05 significance level. Wald Chi-Square tests was used to assess for the interaction terms, with *p* < 0.1 considered significant. We did not include patients with missing data or those lost to follow-up in the regression analyses. Differences in survival between the two groups were compared using the log-rank test. Hazard ratios with 95% confidence intervals and *p*-values are reported. All tests were 2-sided, and statistical significance was defined by *p* < 0.05.

## Results

### Study population

**Fig. 1:**
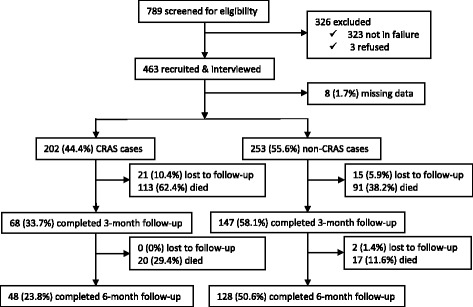
Study Profile

Figure 1 shows the study profile (enrollment, follow-up and survival). During the study period, 463 of the 789 screened patients met the inclusion criteria and were recruited into this study. The baseline demographic characteristics were similar amongst study participants regardless of their CRAS status; however, their baseline clinical and laboratory characteristics displayed a different pattern, Table 1. The mean age of participants was 46.4 ± 18.9 years, and 257 (56.5%) were women. Ninety two point three percent (92.3%) of patients were in New York Heart Association (NYHA) functional class III/IV on admission. A total of 246 (54.1%) participants had a history of hypertension, 67 (14.7%) had diabetes and 31 (6.8%) were HIV infected (Additional file 1).

**Fig. 2:**
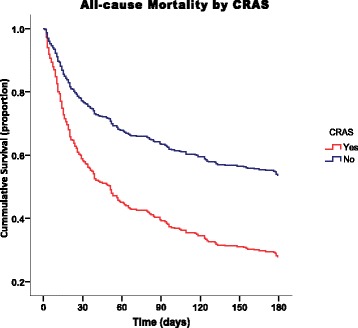
Cox Proportional Hazard Model Survival Curve by CRAS

**Table 1:** Baseline Demographic & Clinical Characteristics of the Study Population

Characteristic	All Patients (*n* = 455)	CRAS (*n* = 202)	Non-CRAS (*n* = 253)	*p*-Value
**Demographics**				
- Age, mean (SD), years	46.4 (18.9)	48.1 (17.8)	45.0 (19.7)	
- Age groups				0.09
< 30	112 (24.6)	42 (20.8)	70 (27.7)	0.09
30–50	140 (30.8)	65 (32.2)	75 (29.6)	0.56
> 50	203 (44.6)	95 (47.0)	108 (42.7)	0.35
- Female sex, no (%)	257 (56.5)	124 (61.4)	133 (52.6)	0.06
- Primary education (%)	319 (70.1)	136 (67.3)	183 (72.3)	0.25
- Self-employment (%)	226 (49.7)	99 (49.0)	127 (50.2)	0.80
- Urban residence (%)	303 (66.6)	132 (65.4)	171 (67.6)	0.61
- Ever smoker (%)	49 (10.8)	22 (10.9)	27 (10.7)	0.95
- Health insurance (%)	97 (21.3)	41 (20.3)	56 (22.1)	0.64
**Vital sign,** mean (SD)				
- Respiratory Rate, breaths/min	24.1 (6.5)	23.9 (6.5)	24.3 (6.5)	0.48
- Heart rate, beats/min	98.5 (22.2)	94.5 (21.0)	101.7 (22.7)	**<0.001**
- Systolic BP, mmHg	132.4 (35.9)	144.7 (40.2)	122.5 (28.5)	**<0.001**
- Diastolic BP, mmHg	82.3 (24.7)	87.2 (26.6)	78.4 (22.3)	**<0.001**
- Oxygen saturation, %	96.3 (7.6)	96.5 (7.6)	96.2 (7.6)	0.64
- NYHA III&IV (%)	420 (92.3)	188 (93.1)	232 (91.7)	0.58
- BMI, kg/m^2^	25.1 (5.2)	25.6 (4.7)	24.8 (5.6)	0.13
**Serum concentration**, mean (SD)				
- Hemoglobin, g/dL	10.3 (3.1)	8.3 (2.3)	9.9 (2.1)^a^	**<0.001**
- Mean Cell Volume, fL	82.9 (9.0)	81.5 (8.4)	84.0 (9.4)	**<0.01**
- Mean Cell Hemoglobin, pg/cell	26.6 (3.6)	25.9 (3.1)	27.1 (4.0)	**<0.001**
- Creatinine, μmol/L	446.3 (679.1)	870.1 (838.2)	278.2 (224.0)^b^	**<0.001**
- Total cholesterol, mg/dl	4.2 (2.2)	5.0 (2.4)	3.6 (1.8)	**<0.001**
- LDL cholesterol, mg/dl	3.2 (2.0)	3.9 (2.1)	2.5 (1.7)	**<0.001**
- HDL cholesterol, mg/dl	1.0 (0.4)	0.9 (0.5)	1.0 (0.4)	0.25
- Sodium, mmol/L	132.0 (8.3)	131.3 (8.8)	132.6 (7.9)	0.9
Hyponatremia (%)	232 (54.7)	112 (59.3)	120 (51.1)	0.09
- Potassium, mmol/L	4.4 (1.3)	4.9 (1.5)	4.1 (1.0)	0.9
Hypokalemia (%)	80 (18.4)	28 (14.5)	52 (21.6)	0.06
Hyperkalemia (%)	130 (30.0)	87 (45.1)	43 (17.8)	**<0.001**
- Calcium, mmol/L	2.1 (0.3)	2.1 (0.3)	2.1 (0.2)	1
Hypocalcemia (%)	169 (48.8)	94 (54.3)	75 (43.4)	**0.04**
- Magnesium, mmol/L	0.9 (0.2)	0.9 (0.2)	0.8 (0.1)	1
Hypomagnesemia (%)	41 (16.1)	14 (11.0)	27 (21.1)	**0.03**
**Comorbid conditions**				
- Hypertension (%)	246 (54.1)	143 (70.8)	103 (40.7)	**<0.001**
- Diabetes (%)	67 (14.7)	42 (20.8)	25 (9.9)	**<0.01**
- HIV (%)	31 (6.8)	18 (8.9)	13 (5.1)	0.11
**Admission Duration**, mean, days (SD)	13.9 (13.0)	14.4 (11.0)	13.5 (14.5)	0.42

^a^ and ^b^ represents anemic (*n* = 129) and renal insufficiency (*n* = 34) subgroups amongst non-CRAS participants respectively

### Prevalence and associated factors for CRAS

Of the 455 participants with complete data, 236 (51.9%) had renal insufficiency and 331 (72.8%) had anemia. We found that 202 (44.4%) of our heart failure cohort had the CRAS. Participants with CRAS displayed a higher likelihood for both end-stage renal disease (ESRD) (eGFR < 15) and the severe form of anemia (Hb < 8 g/dL) compared to their non-CRAS counterparts with renal dysfunction or anemia, (both *p* < 0.001). Comorbid diabetes or hypertension was associated with higher rates of CRAS compared to those free from either condition, (*p* < 0.01 & *p* < 0.001 respectively).

Overall, hypertensive heart diseases (40.9%) was the leading cause of heart failure, followed by cardiomyopathies (26%) and valvular heart diseases (23%). Of the valvular etiology, 82.6% were attributable to rheumatic heart disease. We observed that 114/236 (48.3%) of participants with renal insufficiency were in ESRD and 14 (12.3%) of these were on dialysis. Normocytic normochromic anemias constituted 180/331 (54.4%) of anemias, while the microcytic hypochromic form was found in 107/331 (32.3%) of anemic subjects.

### Primary outcome

During a mean follow-up of 103 ± 75 days, 241/417 (57.8%) participants died, with 21.6% of these fatalities occurring either at home or on the way to a health facility. Patients with CRAS had a significantly higher mortality rate 133/181 (73.5%) compared to those free of CRAS 108/236 (45.8%), (*p* < 0.001), Fig. 2. Mortality rate was observed to increase with worsening of heart failure i.e. 33.3%, 54.2%, and 63.2% in NYHA class II, III, and IV respectively. Presence of anemia and/or renal insufficiency was observed to independently and synergistically increase the mortality rate, Table 2. During multivariate analysis in a cox regression model of 21 potential associated factors for mortality; renal dysfunction (HR 1.9; 95% CI 1.0–3.5; *p* = 0.03), severe anemia (HR 1.8; 95% CI 1.0–3.1; *p* = 0.04), hyponatremia (HR 2.2; 95% CI 1.3–3.7; *p* = 0.004) and rehospitalization (HR 4.3; 95% CI 2.2–8.4; *p* < 0.001) proved to be the strongest factors.

**Table 2:** Mortality Rates (%) by CRAS Triad Components at 30, 90 & 180 Days

	30-day	90-day	180-day
HF	22.7	31.3	39.0
HF + Anemia	24.6	39.7	47.5
HF + CKI	33.3	50.0	55.9
HF + CKI + Anemia	41.4	62.4	73.5

Key: *HF* Heart Failure, *CKI* Chronic Kidney Insufficiency

### Subgroup analysis

**Fig. 3:**
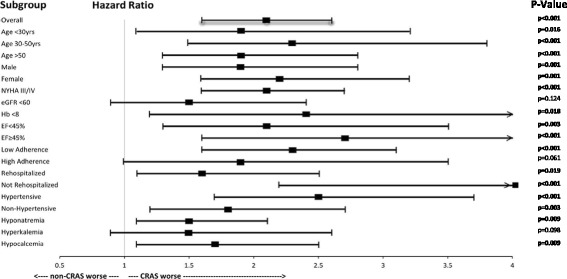
Hazard Ratios for All-cause Mortality by CRAS. This forest plot shows the hazard ratios (*black squares*), 95% CIs (*horizontal lines*), and *p*-values for the interaction between the all-cause mortality and any subgroup variable by CRAS-status

The superior rate of death observed in participants with CRAS was consistent across all major subgroup analyses we conducted, (Fig. 3). Overall, patients with CRAS displayed a two times mortality rate compared to non-CRAS participants, (HR 2.1, 95% CI 1.6–2.6, *p* < 0.001).

## Discussion

The present study shows that CRAS is considerably prevalent and is associated with increased mortality among hospitalized patients with heart failure. These observations are in unison with the previously published data [1–8]. Compared to participants without comorbid renal dysfunction and anemia, patients with CRAS had a 2-fold mortality hazard. Contrary to previous studies, we observed very high mortality rates within a short period of follow-up. These findings were independent of demographic confounders and established risk factors. Over one fifth of the deceased participants died before reaching a health facility. The authors believe this is so because of the weak emergency systems, poor infrastructure and extreme poverty which hinder a timely access to healthcare services in low and medium countries like Tanzania.

The mean age of our heart failure cohort was 46 years. This is a relatively young age compared to the mean of >70 years reported in the western world [9–11]. Such young onset of heart failure has been reported in a number of developing nations and remain a major concern. Hypertension continues to be the leading cause of heart failure in resource limited settings. We observed that hypertensive patients had a doubled risk of having CRAS compared to their hypertension-free counterparts, (RR = 2.1, 95% CI 1.6–2.6, *p* < 0.001).

Renal insufficiency was present in over a half of the study participants. These findings are in consonance with a number of previous studies [12, 13]. Anemia prevalence and severity was found to increase with worsening of renal functions. These findings are in keeping with our current understanding of anemia’s potential in causing renal insufficiency as well as worsening it. Only 12% of patients in ESRD were on dialysis, this is probably due to the remaining high cost of dialysis despite an increasing number of service providers in this setting.

Almost three-quarters of patients in this present study were anemic. The reported anemia rates among heart failure patients have a wide range among studies and our rate falls within [1]. This variability is partly attributable to the use of inconsistent definitions of anemia (i.e. Hb cut-off points) and/or different inclusion criteria for age among studies. In this set-up however the anemia prevalence is reportedly high even in the general population almost certainly due to a significant burden of malnutrition and infectious diseases. We therefore hypothesize that a good number of heart failure patients were primarily anemic even before the onset of heart failure.

Our study has several strengths. First, our diagnosis of heart failure though initially utilized a screening tool (Framingham criteria), the diagnostic confirmation relied on a 2-dimensional echocardiography. Enrolled patients were admitted in a tertiary hospital with a national status receiving cases from the whole country and thus our findings are perhaps generalizable to Tanzania and similar resource limited settings. We reported the all-cause mortality which is a relatively unbiased and the most valid endpoint.

This study was observational with obvious limitations. The absence of serial measurements of hemoglobin and creatinine in this study implies that we missed new cases and trends of progression of anemia and renal insufficiency that occurred during the follow-up period. Moreover, with a single creatinine measurement taken, we were not in a position to qualify whether the renal insufficient participants were in acute or chronic phase.

## Conclusion

The CRAS is considerably prevalent and is associated with an increase of mortality amongst patients with heart failure. Our results suggest that in patients with heart failure; timely, aggressive and collaborative measures to improve renal functions and/or correct anemia might improve survival of heart failure patients. Furthermore, these findings call for guideline committees to revise and/or develop evidence-based recommendations for management of patients with CRAS.

## Additional file

Additional file 1: CRAS study Tanzania - dataset. (XLSX 155 kb)

## Abbreviations

CRAS: Cardiorenal anemia syndrome
EF: Ejection fraction
eGFR: Estimated glomerular filtration rate
ESRD: End stage renal disease
Hb: Hemoglobin
HIV: Human immunodeficiency virus
HR: Hazard ratio
MDRD: Modification of diet in renal disease
NYHA: New York Heart Association
RR: Relative risk
sCr: serum creatinine
